# A Case Study of Tire Crumb Use on Playgrounds: Risk Analysis and Communication When Major Clinical Knowledge Gaps Exist

**DOI:** 10.1289/ehp.7629

**Published:** 2005-08-17

**Authors:** Mark E. Anderson, Katherine H. Kirkland, Tee L. Guidotti, Cecile Rose

**Affiliations:** 1 Department of Community Health Services, Denver Health, Denver, Colorado, USA; 2 Department of Pediatrics, University of Colorado Health Science Center, Denver, Colorado, USA; 3 Association of Occupational and Environmental Clinics, Washington, DC, USA; 4 Department of Environmental and Occupational Health, Mid-Atlantic Center for Child Health and the Environment, School of Public Health and Health Sciences, George Washington University Medical Center, Washington, DC; 5 Departments of Medicine/Preventive Medicine and Biometrics, National Jewish Medical and Research Center, Denver, Colorado, USA

**Keywords:** children, environment, PEHSU, playground, recycled tire, risk communication, tire crumb

## Abstract

Physicians and public health professionals working with the U.S. Environmental Protection Agency’s Region 8 Pediatric Environmental Health Specialty Unit (PEHSU) received several telephone calls requesting information regarding the safety of recycled tire crumb as a playground surface constituent placed below children’s play structures. There were no reported symptoms or adverse health effects in exposed children. The literature available on the safety and risk of exposure to crumb rubber constituents was limited and revealed no information quantifying exposures associated with product use. Callers were informed by the PEHSU that no evidence existed suggesting harm from intended use of the product, but gaps in knowledge about the product were identified and communicated. Here the case of crumb rubber on playgrounds is used as a model to present an approach to similar environmental medicine questions. From defining the question, to surveying traditional and nontraditional resources for information, synthesis of findings, and risk communication, the case provides a model to approach similar questions.

## Case Presentation

The U.S. Environmental Protection Agency’s (EPA) Rocky Mountain region (Region 8) Pediatric Environmental Health Specialty Unit (PEHSU) received three telephone inquiries from parents concerned about health risks to children exposed to a recycled tire crumb product used as a soil additive, or “amendment,” on school playgrounds. A school district in the region had applied the product, which is made from recycled automobile tires, under outdoor play structures as an alternative to sand or wood chips. The crumbled tire amendment had the appearance of very small ball bearings or large, round grains of sand. One of the parents reported finding fibers in her child’s hair each day after school, and noted similar fibers in the lint collector of the family’s clothes dryer. The callers also reported seeing as the children played a visible haze in the air above the playground that used the tire crumb. No odor was reported with this haze, nor was the incident further characterized by time of day, moisture, or dust. The callers were aware of no illnesses among school children associated with exposure to the product, and specifically knew of no new or worsened respiratory illnesses such as asthma. None of the children of these concerned parents had reported skin, eye, lung, or mucous membrane irritation symptoms. One of the callers had attempted extensively to investigate the product and confessed to difficulty in her dealings with the manufacturer.

## Discussion

The callers’ question regarding the safety of tire crumb for use on children’s playgrounds is appropriate, and the case highlights many of the difficulties that care providers and health advisors encounter in daily practice. Knowledge gaps are more typical than is established science, especially when children are the exposed population. This case report exemplifies the pathway that care providers can follow from the concerned parent’s question to risk communication regarding the exposure. Important steps along the pathway include defining the question, searching the literature for published information, searching for information from nontraditional sources such as the manufacturer, looking to relevant governmental agencies for information, synthesizing the information gathered, and finally, offering a summary of the information with recommendations to the parent.

The approach to the callers’ question begins by defining the question. The stated concern relates to use of a loose, crumbled product made from used tires. Children playing on tire crumb could potentially be exposed by ingestion of the product directly, by ingestion of surface water runoff through the product, by inhalation of dust, or by skin contact with the material or surface water runoff. An alternative crumb rubber product embeds the loose product into a resin to form a tile, which is placed over a hard surface and locked together with other tiles. Nonplayground uses include as an asphalt additive in road building and as an aggregate in concrete. Tire crumb contributes to the strength of concrete, and the product is reportedly lighter in weight than typical concrete (Pierce and Blackwell 2003). The Region 8 Agency for Toxic Substance Disease Registry (ATSDR) has done specific work suggesting that smokestack emissions produced by burning tire crumb to generate electricity are comparable with that of coal, with some minor differences (Willis et al. 2003). Conceivably, these industrial exposures should not be applicable to children. The many references to use of tire crumb were irrelevant to our central question regarding safety with its use on playground surfaces.

To examine further the known risks to children from exposure to the playground product, we turned to traditional published scientific literature and the network of PEHSUs in the United States. One study, done by investigators working in Alberta (Birkholz et al. 2003), examined the human and ecosystem hazard presented by tire crumb using *in vitro* mutagenicity assays. The associated hazard analysis suggested that the risk associated with playground use was very low. Toxicity to all of the aquatic organisms tested was observed in the fresh aqueous extract, but activity disappeared with aging of the tire crumb for 3 months in place on the playground. The investigators concluded that the use of tire crumb in playgrounds results in minimal hazard to children and the receiving environment, assuming intended use of the product, such as exclusive outdoor use and the presence of no solvents other than water. Regarding our central question of potential harm to children, the published literature contained some information about the product, including an *in vitro* toxicity model, but traditional published resources and a network of environmental health experts could not establish the product’s safety in use with children.

We then turned to additional resources for information: manufacturers and governmental resources such as the U.S. EPA and the ATSDR. Several states have published information regarding recycled tires and tire disposal (Arizona Department of Environmental Quality 2002; Moulton-Patterson et al. 2003; Texas Commission on Environmental Quality 2003). The Consumer Product Safety Commission had no information available on the crumb rubber product. However, after surveying these resources, we knew little more about the crumb rubber product in its playground application. A last search for information used the Google online search engine (http://www.google.com). This yielded multiple industry, federal, state, and local websites with information on the use of recycled tires and tire disposal. These resources failed to address our central question regarding children’s contact with the crumb rubber product on playgrounds.

Some literature was informative regarding use of the product and potential dangers. The crumb rubber on playgrounds case may typify environmental medicine cases where published information is available but not necessarily relevant. Research suggests that work with heated asphalt containing recycled tire crumb may expose workers to carcinogenic polycyclic aromatic hydrocarbons including benz[*a*]anthracene, chrysene, and their methylated derivatives (Watts et al. 1998). The tire crumb product has been studied for safety in its intended use as a playground surface amendment using the methods of risk assessment, genotoxicity assays, and ecotoxicity assays. The investigators concluded that it probably would not represent an exposure hazard for children or risk to the environment (Birkholz et al. 2003). An epidemiologic study to validate the findings on human risk has not been feasible.

Compiling the information gathered into a statement of potential risk requires a balanced presentation of benefits and problems involved with a given product. As for the tire crumb product, several potential advantages exist in its use as a playground surface amendment. Although most discarded tires are placed into landfills to degrade slowly, economic use of tire crumb diverts old tires from landfills and piles where they present serious hazards. Stockpiled tires in landfills can contribute to fires that are difficult to extinguish, releasing combustion products (e.g., benzene, other volatile organic hydrocarbons, and dioxins) into the air. The hollow structure of a tire creates a breeding space for human disease vectors. Advantages are that the crumb product is lightweight and cost-effective according to school district users. The manufacturer claims that the product has a superior degree of cushioning against falls, the main purpose of its use below play structures. Direct application is simple and cheap, much like that of sand: Simply shovel it into place.

Despite potential advantages in terms of injury prevention and waste recycling, the use of recycled tire crumb products on playgrounds has had little health investigation. The major unresolved concern is the potential for latex allergy with short-term dermal exposure. Latex is a known airway and dermal sensitizer, but the vulcanized chemistry of tire manufacture should destroy these allergens. Latex allergens have been identified as components of urban air, and the potential risk of tire crumb must be distinguished from the potential risk of “tire dust,” which has been considered a possible hazard in urban air pollution. A study of particulate air pollutants in Denver, Colorado, found black respirable particulates that were identified as airborne tire fragments (Williams et al. 1995). Williams et al. (1995) suggest that these respirable “tire dust” particles may contribute to the pathogenesis of lung diseases related to air pollution. Whether respirable particles are created during regular use of the tire crumb product requires further investigation, given that a high amount of energy is required to create smaller crumb rubber particles. Reports of haze while children played on the applied product may or may not be related.

The process of risk communication should include limited use of vernacular and, most important, an open offer to maintain communication. Success should not be measured in “closing the case” but in gaining the confidence of the callers so that information continues to flow in a meaningful and productive manner. This is important not only for care providers who may encounter these difficult questions, but also for governmental and nongovernmental entities to maintain productive communication with concerned callers. In our crumb rubber case, the callers expressed frustration in communications with the manufacturer. If true, this represents an error on the part of the manufacturer because this only creates enmity, may fuel further questioning, and does not promote a meaningful process of inquiry.

The pathway from question to risk communication is lengthy, time consuming, and is not practical for a typical provider to follow, unless specific expertise or interest exists. The crumb rubber case involved input from the providers working with the Region 8 PEHSU, the national PEHSU network, Region 8 EPA, and ATSDR, the Consumer Product Safety Commission, and the manufacturer. Searching the literature was time-consuming and did not yield an answer to the question. Although time-consuming and sometimes not fruitful, the process is an important one. Any initiatives that join together professionals with specific expertise and care providers who encounter the initial questions are valuable in that they allow a quick inquiry and should be used extensively. The case demonstrates the wisdom of the PEHSU network.

## Conclusion

**Figure 1:**
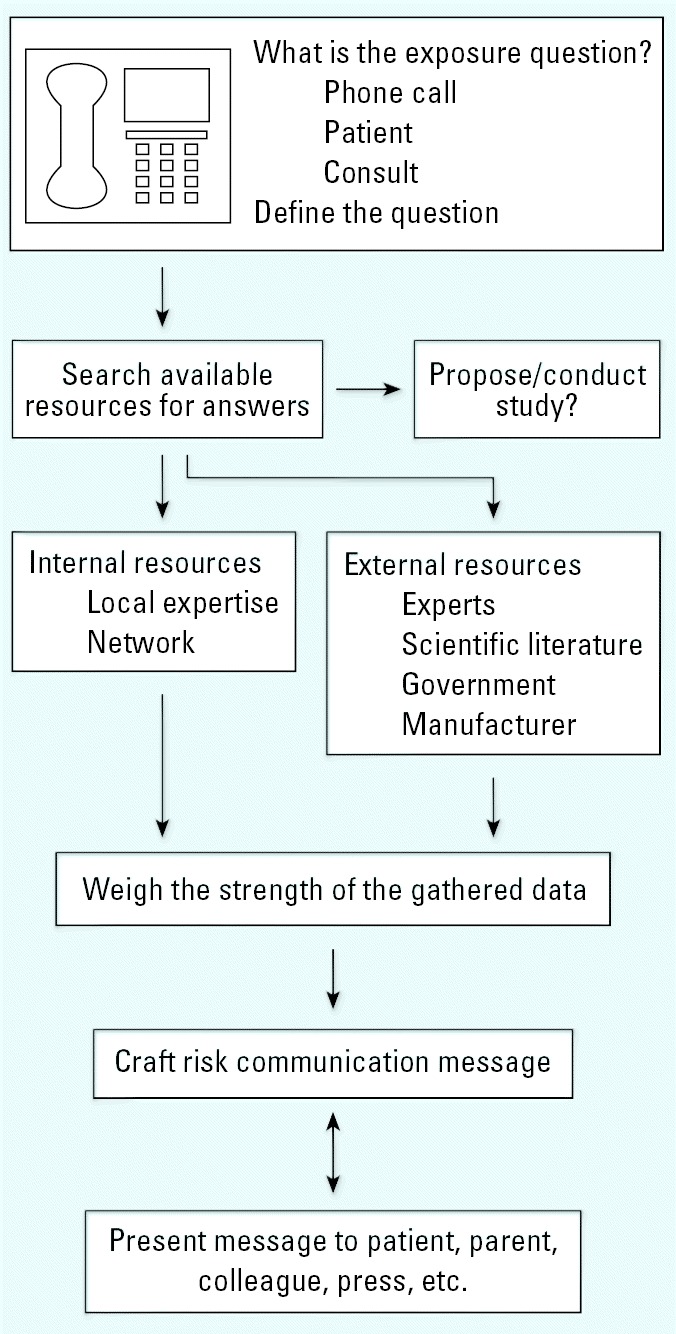
The process of risk communication. Crafting and communicating a message regarding risk with a specific exposure begins by defining the exposure, searching traditional and nontraditional resources, weighing the gathered information, crafting the message, and maintaining an open channel of communication around the potential exposure.

Environmental health professionals commonly encounter questions regarding the safety of a particular product or substance. Although the literature may yield some answers, knowledge gaps are common, and networks such as the national PEHSUs can be invaluable resources in the search for information. We present here a case involving use of crumb rubber tire as a surface amendment on playgrounds as a model case where the published literature did not contain the needed answers. We demonstrate a process, also shown in Figure 1, from defining and researching the question to risk communication with concerned callers or parents.

No published information is available specifically regarding exposure to crumb rubber constituents from use of the product on playgrounds. The research by Birkholz et al. (2003) addressed many of the potential concerns but did not include evaluation of actual playground use. The product has several obvious advantages including the useful recycled use of a product that is otherwise discarded and improved mechanical safety under playground equipment. Some published information was available discussing use of the product in asphalt installation, for example. Risks may exist in working with the product, but the question regarding hazards posed to children playing on the amended playgrounds is left unanswered. Clearly, more investigation is needed, and efforts such as those by the California Integrated Waste Management Board to look at the use of tire crumb and the potential for release of respirable particles are timely and welcome.

Communication with callers or parents regarding the information and study on the product and the development of a message regarding potential risk is the final step in the investigation process. The risk message should include a straightforward statement regarding what is known and offer an open channel of communication regarding continued concern with the product. The role for entities such as the regional PEHSUs is critical to this process because it requires time and the special expertise that may not be directly available to typical care providers. Providers encountering children who may be suffering illness related to exposure to products such as tire crumb should involve their environmental health colleagues and federal, state, and local resources extensively.
